# A complete circular genome of ΦX174 host *Escherichia coli* C122

**DOI:** 10.1128/mra.00061-25

**Published:** 2025-04-29

**Authors:** Victoria A. Sharp, Siobain Duffy

**Affiliations:** 1 Microbial Biology Graduate Program, Rutgers University 5970, New Brunswick, New Jersey, USA; 2 Department of Ecology, Evolution, and Natural Resources, School of Environmental and Biological Sciences, Rutgers University, New Brunswick, New Jersey, USA; Department of Biology, Queens College, Queens, New York, USA

**Keywords:** Enterobacteriaceae, *Escherichia coli*, phiX174

## Abstract

*Escherichia coli* C122 and the bacteriophage that uses it as the standard laboratory host (ΦX174) make up a dream team of model microorganisms. This ΦX174 host is the second complete, published genome of *E. coli* C122, and is ~1% different from the other, justifying that it is another strain.

## ANNOUNCEMENT

*E. coli* C122 and ΦX174 are an established pathosystem and arguably one of the best-understood host-virus systems (1). The ~4.6 Mb circular double-stranded DNA genome of *E. coli* C122 was assembled from Nanopore R10.4.1 flow cell reads.

The bacterial strain, a known host for ΦX174, was acquired from Bentley Fane (University of Arizona), who originally obtained it from Masaki Hayashi, the same “BTCC 122” isolate used by Sinsheimer for his ΦX174 work (2). Glycerol stock cultures with 40% glycerol were stored at −70°C. A loopful of bacterial cells from one freezer stock was streaked onto TK agar plates and incubated for 24 h at 37°C. A single colony was selected to inoculate 10 mL of TK broth and be grown overnight at 37°C in a shaking incubator at 110 rpm. In addition, 1,000 µL of the culture was added to a microfuge tube and centrifuged at 3,000 rpm for 10 min. The supernatant was poured off, and the pelleted *E. coli* C122 was resuspended in Zymo DNA/RNA Shield and shipped to Plasmidsaurus where the DNA was extracted using the Zymo Quick-DNA Miniprep Plus Kit and sequenced using long-read Oxford Nanopore technology (ONT). This involved the construction of an amplification-free long-read sequencing library on unsheared DNA with v14 library prep chemistry, using R10.4.1 flow cells (managed by MinKNOW v23.07) to sequence primer-free reads. Default parameters were used for all software unless otherwise specified. The worst 5% of reads were removed with Filtlong v.0.2.1. The remaining reads were roughly assembled with Miniasm v0.3, assembled with Flye v2.9.1 (parameters for high-quality ONT reads), and polished with Medaka v1.8.0. Genome completeness was assessed with Bandage v0.8.1, and circularity was further checked by BLASTing a concatenation of ~100 bases at the ends of the fasta file (other *E. coli* genomes had 100% query cover with up to 99.62% identity).

With 99× coverage, one contig was produced alongside 234,884 reads, 4,554 annotated genes, and 4,606,320 base pairs. GC content was 50.96% and N50 was 8,861 bp. The bacterial genome was uploaded to GenBank, annotated using NCBI’s PGAP, and has the accession number CP170128.

CJ Bioscience’s online Average Nucleotide Identity (ANI) calculator was used to determine ANI between CP170128 and the other complete C122 genome (CP029371 [3]) as 99.02%. This supports the two sequenced C122 isolates being different strains (4). CP029371 was sourced from the DSMZ, from a strain “no longer recommended as a host for ΦX174,” https://www.dsmz.de/collection/catalogue/details/culture/DSM-4860 . However, our strain is nearly identical (ANI of 99.99%) to the sequence of NCTC 122 (LT906474), which is not listed as an *E. coli* C in GenBank. Sinsheimer, and later Hayashi, denoted their strains BTCC 122 (not NCTC 122), but the high identity of the sequences indicates that those who received “BTCC 122” from Nikolai Bulgakov (5) were receiving a strain closely descended from the NCTC 122 isolate, deposited by the Lister Institute in 1920. There are 21 base substitutions (12 transitions, nine transversions) between our strain and NCTC 122 and several indel mutations, the largest of which are listed in Table 1. Most of the difference in length between the longer NCTC122 sequence and our sequence is due to two large deletions, and our C122 has had an additional copy of an existing transposon added to its genome (there are 20 IS3-like transposases in our C122 sequence).

**TABLE 1:** Larger insertion/deletion mutations differentiating our C122 (CP170128) from NCTC 122 (LT906474)

Indel present in	Length	Location in genome	Predicted function of genes
CP170128.1	186	3,484,841–3,485,026	N-acetylmuramoyl-L-alanine amidase AmiC
CP170128.1	1,224	1,874,995–1,876,218	IS3-like element ISSen4 family transposase
LT906474.1	9,820	4,569,066–4,578,877	Ribose transport proteins
LT906474.1	11,105	2,199,481–2,210,585	Electron transport proteins

## Data Availability

The genome data are found in GenBank under accession number CP170128 and raw reads under SRA accession number PRJNA1160296.
